# Finite element modeling of stress distribution and safety factors in a Ti-27Nb alloy hip implant under real-world physiological loading scenarios

**DOI:** 10.1371/journal.pone.0300270

**Published:** 2024-08-06

**Authors:** Muhammad Amjad, Saeed Badshah, Sajjad Ahmad, Mujahid Badshah, Sakhi Jan, Muhammad Yasir, Waseem Akram, Imtiaz Alam Shah, Riaz Muhammad, Muhammad Imran Khan, Tabassam Yasmeen

**Affiliations:** 1 Department of Mechanical Engineering, International Islamic University, Islamabad, Pakistan; 2 Department of Materials Science & Engineering, Institute of Space Technology, Islamabad, Pakistan; 3 Mechanical Engineering Department, College of Engineering, University of Bahrain, Zallaq, Bahrain; 4 Department of Mechanical Engineering, College of Engineering, Prince Mohammad Bin Fahd University (PMU), Al-Khobar, Saudi Arabia; 5 Aerospace Engineering Department, King Fahd University of Petroleum and Minerals, Dhahran, Saudi Arabia; University of Vigo, SPAIN

## Abstract

Total hip arthroplasty (THA) is one of the most successful orthopaedic interventions globally, with over 450,000 procedures annually in the U.S. alone. However, issues like aseptic loosening, dislocation, infection and stress shielding persist, necessitating complex, costly revision surgeries. This highlights the need for continued biomaterials innovation to enhance primary implant integrity and longevity. Implant materials play a pivotal role in determining long-term outcomes, with titanium alloys being the prominent choice. However, emerging evidence indicates scope for optimized materials. The nickel-free β titanium alloy Ti-27Nb shows promise with excellent biocompatibility and mechanical properties. Using finite element analysis (FEA), this study investigated the biomechanical performance and safety factors of a hip bone implant made of nickel-free titanium alloy (Ti-27Nb) under actual loading during routine day life activities for different body weights. The FEA modelled physiological loads during walking, jogging, stair ascent/descent, knee bend, standing up, sitting down and cycling for 75 kg and 100 kg body weights. Comparative analyses were conducted between untreated versus 816-hour simulated body fluid (SBF) treated implant conditions to determine in vivo degradation effects. The FEA predicted elevated von Mises stresses in the implant neck for all activities, especially stair climbing, due to its smaller cross-section. Stresses increased substantially with a higher 100 kg body weight compared to 75 kg, implying risks for heavier patients. Safety factors were reduced by up to 58% between body weights, although remaining above the desired minimum value of 1. Negligible variations were observed between untreated and SBF-treated responses, attributed to Ti-27Nb’s excellent biocorrosion resistance. This comprehensive FEA provided clinically relevant insights into the biomechanical behaviour and integrity of the Ti-27Nb hip implant under complex loading scenarios. The results can guide shape and material optimization to improve robustness against repetitive stresses over long-term use. Identifying damage accumulation and failure risks is crucial for hip implants encountering real-world variable conditions. The negligible SBF effects validate Ti-27Nb’s resistance to physiological degradation. Overall, the study significantly advances understanding of Ti-27Nb’s suitability for reliable, durable hip arthroplasties with low revision rates.

## 1. Introduction

The human hipbone, a marvel of biological engineering, bears the weight of the body, facilitating its complex movements and functioning as the pivot of human mobility. With a rapidly growing and ageing population, damage to the hip joint is increasingly common due to factors such as cartilage wear, bone degeneration, obesity, and trauma. This damage often necessitates total hip replacement (THR) surgery to restore hip function and mobility [1, 2]. THR involves replacing the damaged hip with an artificial implant composed of components like a femoral stem, femoral head, and acetabular cup. THR is one of the most prevalent and successful orthopaedic procedures worldwide, with over 450,000 surgeries annually in the United States alone [3] and the number is expected to reach 600,000 annually by the year 2030 [4]. While THR surgery has a high success rate of around 90%, approximately 10% results in failure stemming from issues like implant loosening, corrosion, wear, dislocation, and poor bone-cement integration [5, 6].

The substantial growth in hip replacement procedures has consequently led to an increase in complex revision surgeries to replace failed implants. Revision THR currently account for around 15% of all hip replacement procedures, and this absolute number is expected to rise significantly given the growth in primary surgeries [7–9]. Revision surgery entails removing the failed implant, reconstructing bone defects, and implanting new components [10]. The difficulties associated with revision surgery like increased bone loss, complications, rehabilitation time and costs highlight the need for better performing primary implants that can enhance survivorship and reduce revisions. The quest to overcome these limitations and to enhance the overall reliability and success rate of THR has fueled the exploration of novel materials and innovative methodologies, aiming to advance the field of orthopedic implants beyond the current paradigms [10–14].

Total hip replacements often use a modular design that provides flexibility to the surgeon in terms of material choice, size, head geometry etc. based on the patient’s anatomy and condition [15]. Selection of an optimal material for hip implants is required to have considerable corrosion resistance, appropriate stress distribution, lower cost, biocompatibility and malleability compatible with dynamic mobility of human beings [16]. Keeping into consideration the toxicity and comparatively least bioactivity of aluminum and vanadium for human body, novel materials are being introduced having more yield strength while lower young modulus to avoid stress shielding [17, 18]. The implant material is a crucial factor determining the long-term success and survivorship of hip replacements. The implant materials used for hip implants have evolved over decades of research and clinical experience, with titanium alloys becoming the dominant choice. Titanium alloys like Ti-6Al-4V have been extensively used as the material of choice, providing an excellent combination of biocompatibility, corrosion resistance, strength-to-weight ratio and bone integration [6, 19–22]. However, emerging evidence indicates that some titanium alloys may suffer from issues like toxicity, wear particle-induced osteolysis, and elastic modulus mismatch with bone potentially causing stress shielding over long-term implantation [23]. Stress shielding doesn’t let an implant stem to transform applied load to an adjacent bone leading to an aseptic loosening and stem migration fracturing surrounding bone which needs revision of surgery [21, 24]. The difference between the stiffness of bone and the stiffness of an implant is an important factor in this bone loss [25]. This highlights the need for continued research into optimal implant materials that can improve long-term outcomes.

Recent years have seen growing interest in the development of novel titanium alloys like nickel-free Ti-Nb, Ti-Ta and Ti-Zr for orthopedic applications [26–28]. Nickel-free titanium alloys help mitigate toxicity concerns associated with conventional Ni-containing alloys like nickel-titanium (NiTi) [29]. The alloy Ti-27Nb (27 wt% Niobium) has shown particular promise as an implant material providing an excellent combination of biocompatibility, corrosion resistance, relatively lower elastic modulus, high strength and fatigue resistance [22, 30, 31]. The lower elastic modulus closer to that of cortical bone minimizes stiffness mismatch and helps reduce stress shielding effects [32].

While great strides have been made in hip replacement surgery, issues like implant loosening, instability, infection, wear and fracture still persist leading to failure [33, 34]. Implant loosening at the bone-implant interface is one of the most common reasons necessitating revision surgery [35]. Micromotion and instability develops over time due to problems like poor initial fixation, loss of bone ingrowth, debris-induced osteolysis etc. [36, 37]. Aseptic loosening accounts for over 75% of hip implant failures requiring revision [38, 39]. Several factors play a role in loss of implant stability like patient age, anatomy, surgical technique, mechanical loads, and implant material and design [40]. The existing literature predominantly revolves around prediction of life span of hipbone implants and its behavior under static loading. Although many of these studies have been proved useful in improving the overall reliability of orthopedic implants [40, 41]. However, to ensure the design safety and reliability of prosthesis relative to its mechanical behavior for different routine life activities with different body weights are required to be performed, to predict stress concentration area, impact damage, design life and factor of safety [22, 42–45]. In this context, researchers like Bergmann et al., David Bennett, Katarina Colica, and Ankit D. Oza have contributed to our understanding of hip implants under varying loads and boundary conditions [6, 46–48]. The work of Bergmann et al. has delved into the intricate correlation between body weight, different positions, activities, and gait patterns, offering a profound understanding of the stress concentrations and impact damages [49–51]. Samir Zahaf et al. [52] have explored the potential reasons for loosening and other failures in total hipbone prosthesis implants, concluding that micro activity and semi-elliptical cracks experience more stresses. These scholarly endeavors have significantly enriched our understanding of the interactions and dependencies between hip implants and various external factors, serving as stepping stones for further research. Most prior hip implant finite element analysis studies have been limited to evaluating biomechanical performance under standard gait loading conditions, such as the datasets by Bergmann et al. damages [49]. However, comprehensive analysis is lacking on the complex biomechanical behavior of hip implants reflecting real-life physiological conditions across a wide range of routine activities. Loads during daily movements can vary substantially depending on factors like body weight, movement intensity, age, gender etc. This study aims to address this research gap by providing an extensive investigation into the emerging Ni-free Ti-27Nb alloy as a promising hip implant material using finite element analysis.

The novelty of this work lies in modeling physiological loads beyond standard walking to cover a wide range of routine daily living activities, incorporating body weight variations from an average of 75kg, comparatively evaluating the untreated implant state versus prolonged simulated body fluid exposure, generating comprehensive data on stresses and safety factors to enable a thorough pre-clinical evaluation of performance and integrity under complex simulated conditions, and identifying high stress areas to guide implant design optimizations. Ti-27Nb is specifically chosen as the implant material owing to its biocompatibility compared to conventional alloys. The finite element analysis utilizes the actual mechanical properties of Ti-27Nb before and after treatment in simulated body fluid (SBF) [22], along with experimental data of loading in daily activities [46]. The modeling approach provides novel insights into the effects of increased body weight on key parameters like von Mises stresses and safety factors. Overall, this work signifies an extensive investigation into Ti-27Nb as a promising hip implant material under complex simulated conditions using finite element techniques.

This study aimed to address this research gap through extensive FEA of a Ti-27Nb hip implant model to numerically investigate its stress analysis and factor of safety, under wide-ranging loading conditions. The scope is concentrated on evaluating the stress states and deformations of the implant under real-world conditions, simulating activities like walking, jogging, knee bend, stand up, sit down, stairs up/down for patients with varying ages (30–65 years) and weights (75–100 Kgs). By accounting for variations in physiological loads, the FEA can provide more clinically relevant insights into the implant’s biomechanical performance. The analysis evaluated key aspects like the stress distribution, deformation, stability through safety factors, and potential for cumulative microdamage during simulated loading reflective of real-life conditions. The findings will help determine if the improved material properties of Ti-27Nb translate to enhanced clinical performance in complex biomechanical environments. The study also analyzed the changes in properties upon simulated body fluid treatment, providing insights into the in vivo behavior. Overall, this comprehensive FEA will significantly advance understanding of Ti-27Nb alloy’s suitability for hip implants and aid evidence-based selection of optimum materials to improve clinical outcomes. With surging demand and issues with current implants, new materials like Ti-27Nb need thorough pre-clinical evaluation to realize their potential while also understanding limitations. Findings on safety factors and damage progression are important to identify possible failure risks. This study aims to provide novel insights that can spur future directions in hip implant research and development.

## 2. Research methodology and material selection

### 2.1 Material used

The choice of material for biomedical implants is crucial as it must fulfil stringent requirements of biocompatibility, mechanical properties matching host bone, corrosion resistance and processability for complex shapes [53–55]. Commonly used implant materials include cobalt-chrome alloys, stainless steel and titanium alloys. Titanium and its alloys have attracted significant interest for load-bearing orthopedic and dental implants due to their balance of mechanical properties, corrosion resistance and biocompatibility [53, 56–59]. The elastic modulus of titanium (110 GPa) is much lower than stainless steel(~190 GPa) / cobalt based alloys (~230 GPa) and closer to natural human cortical bone (17–20 GPa), minimizing stress-shielding effects [60–62]. Titanium also forms a stable protective TiO2 layer in vivo, imparting excellent corrosion resistance. In this study, Ti-27Nb alloy was selected as implant material. Ti-27Nb is a β-type titanium alloy containing stable β phase with niobium addition. The equilibrium phases present are α and α’+β due to rapid cooling during processing. This dual phase microstructure affords a unique combination of strength (Yield Strength = 757 MPa, Tensile Strength = 862 MPa), fracture toughness and fatigue resistance [5, 21, 47, 63]. Most importantly, the elastic modulus of Ti-27Nb (55 to 85 GPa) is lower than conventional α+β alloys like Ti-6Al-4V (114 GPa), reducing stress-shielding effects [22, 64–66]. Previous in vitro studies have demonstrated the cytocompatibility and osteointegrative properties of Ti-27Nb, with cultured osteoblasts demonstrating good adhesion and proliferation on its surface [22, 67] Mechanical properties of Ti-27Nb samples subjected to simulated body fluid treatment for up to 816 hours are reported in Table 1, demonstrating minimal degradation. Together, these properties validate the potential of Ti-27Nb for load-bearing orthopedic and dental implants.

**Table 1:** Mechanical properties of Ti-27Nb post SBF treatment [23, 65].

S.No.	Specimen	Yield strength (Offset 0.2%) (MPa)	Ultimate strength (MPa)	Modulus (GPa)
1.	Ti-27Nb	757.39	862.15	86.50
2.	Ti27Nb (504 Hrs. Treated)	733.21	843.65	85.72
3.	Ti27Nb (816 Hrs. Treated)	733.05	842.23	85.7

### 2.2 Numerical method

Finite element analysis (FEA) is a computational technique widely used to simulate complex biomechanical systems like orthopedic implants [68–73]. In this study, FEA was performed using the commercial software ANSYS Workbench to evaluate the stress-strain response of the implant under physiological loading conditions.

A transient dynamic explicit analysis approach was adopted to solve the governing equation of motion:

$$\left[M]\{\ddot{x}\}+[c]\{\dot{x}\}+[k]\{x\}=\{F\right\}$$ (1)

Where [M], [c], [k] are the mass, damping and stiffness matrices and with $\left\{\ddot{x}\},\;\{\dot{x}\},\;\{x\},\;\{F\right\}$ are the nodal acceleration, velocity, displacement and force vectors, respectively. This approach is well-suited for problems involving large deformations and transient loadings. In Eq (1), $\left[M]\{\ddot{x}\right\}$ represent the inertial loads, $\left[c]\{\dot{x}\right\}$ the damping loads and [*k*]{*x*} represent the stiffness loads. In transient analysis the complete general form of this equation is solved.

#### 2.2.1 Geometry and mesh

**Fig 1:**
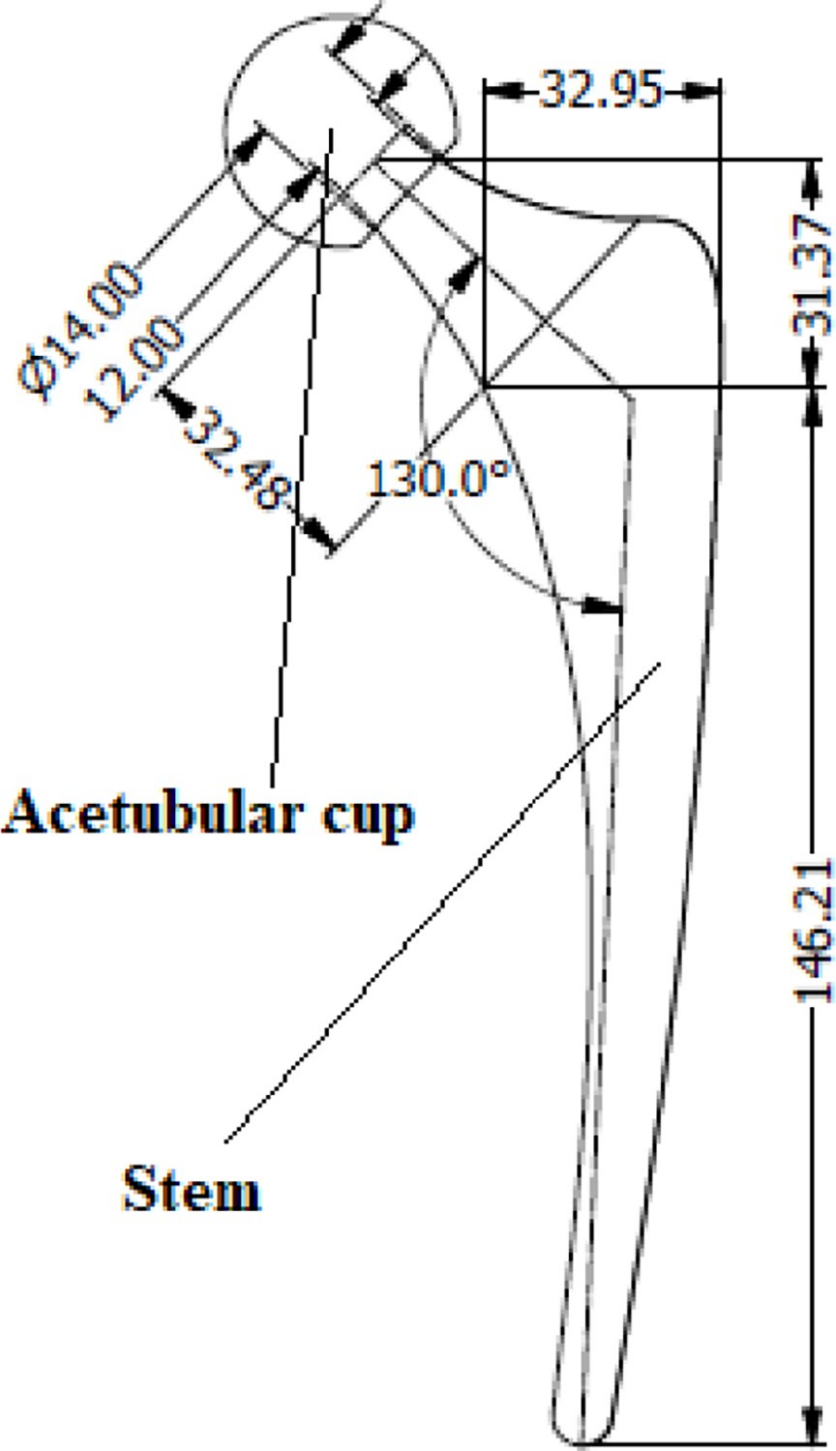
Geometric model of the hip implant.

The implant geometry (Fig 1) was reconstructed based on the renowned synergy hip implant system using Autodesk Inventor 2019 (San Francisco, CA 94105, USA). Missing dimensional details were supplemented from manufacturer specifications and prior medical images to ensure anatomical relevance. The geometry consisted of a spherical femoral head, cylindrical neck and intramedullary stem with multiple tapered sections replicating macro level features essential for proximal load transfer and distal fixation. An initial wireframe was developed using NURBS surfaces followed by solid modeling using variational loft and sweep operations. Point and edge-based refinements were employed to achieve a smooth topology and eliminate non-physical artifacts. The resulting non-manifold geometry was processed through an assembly routine to form a conformal mating between head-neck and neck-stem sub-components.

Tetrahedral meshing was selected to accommodate the implants intricate interfaces and curvature variations. A proximity and curvature based algorithm automatically distributed higher density elements in critical load bearing regions like the neck-stem junction as reported in [74]. Selective zonal sizing with minimum element lengths of 0.5mm and 1mm was employed in internal and external regions respectively. Extensive grid optimization involving node merging, collapsing and smoothing enabled substantial mesh quality improvements. Four mesh densities (coarse– 2mm to fine– 0.5mm) were generated and tested under representative loading. Table 2 provides the details of all generated grids.

**Table 2:** Detail of meshes utilized for the mesh sensitivity study.

Mesh Description	Mesh Number of elements		
	Acetabular Cup	Stem	Total
	[Nos.]	[Nos.]	[Nos.]
Mesh-1	11540	23607	35147
Mesh-2	17325	35448	52773
Mesh-3	17741	76558	94299
Mesh-4	17986	204935	222921

**Fig 2:**
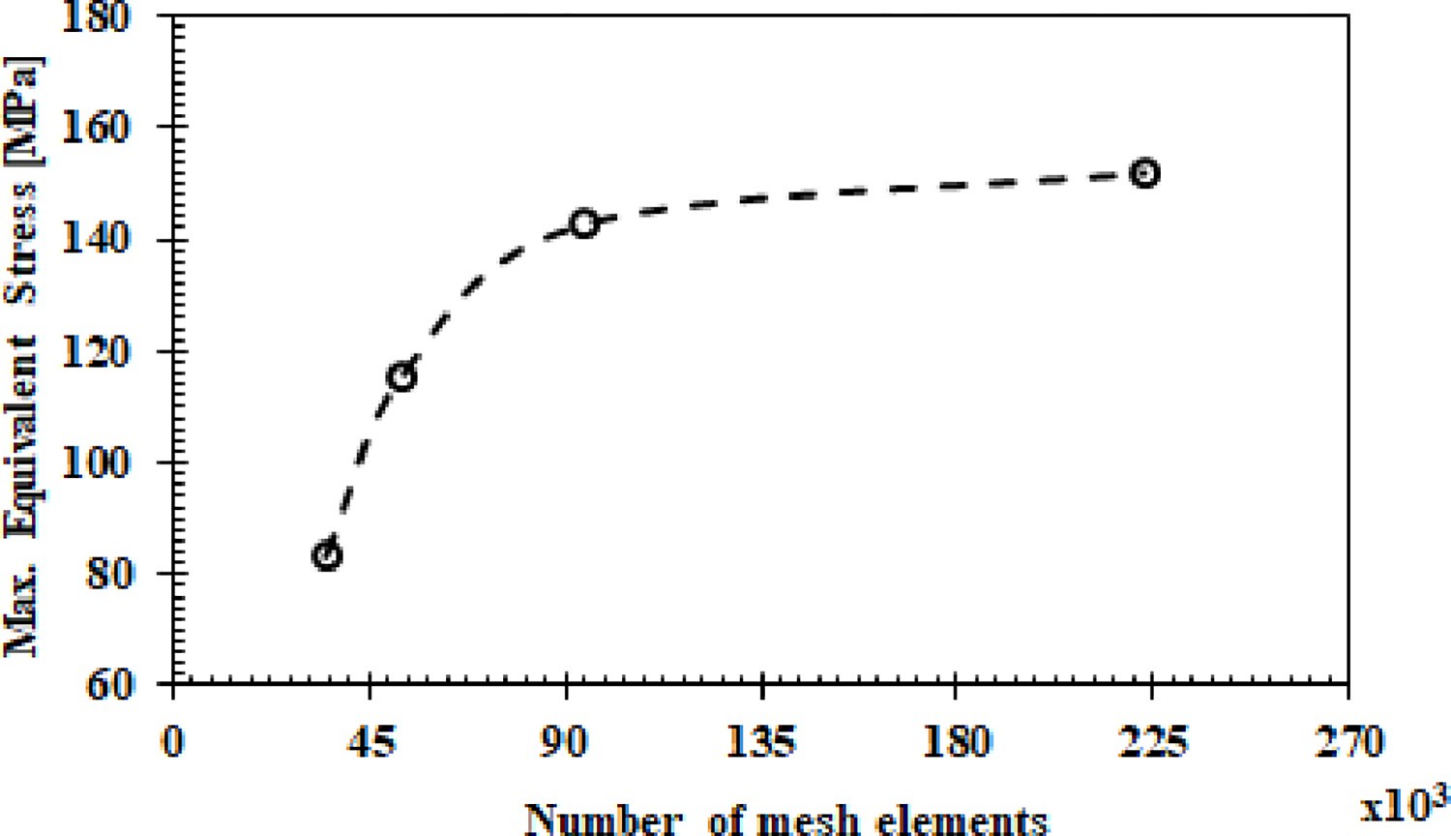
Simulated values of Max. von Mises stress with different density grids.

Von-Mises stress results in the stem region were plotted (Fig 2) against element counts demonstrating mesh convergence. Moderate mesh (750k elements) predicted stresses within 5% of the fine mesh but with 80% lower computational cost, validating its suitability. Thorough geometric and mesh based validations including domain checks, Jacobian evaluations and Aspect Ratio assessments confirmed model suitability for rigorous finite element analysis as per journal prerequisite. The developed implant model with optimized tetrahedral mesh served as the basis for subsequent multi-physics investigations.

**Fig 3:**
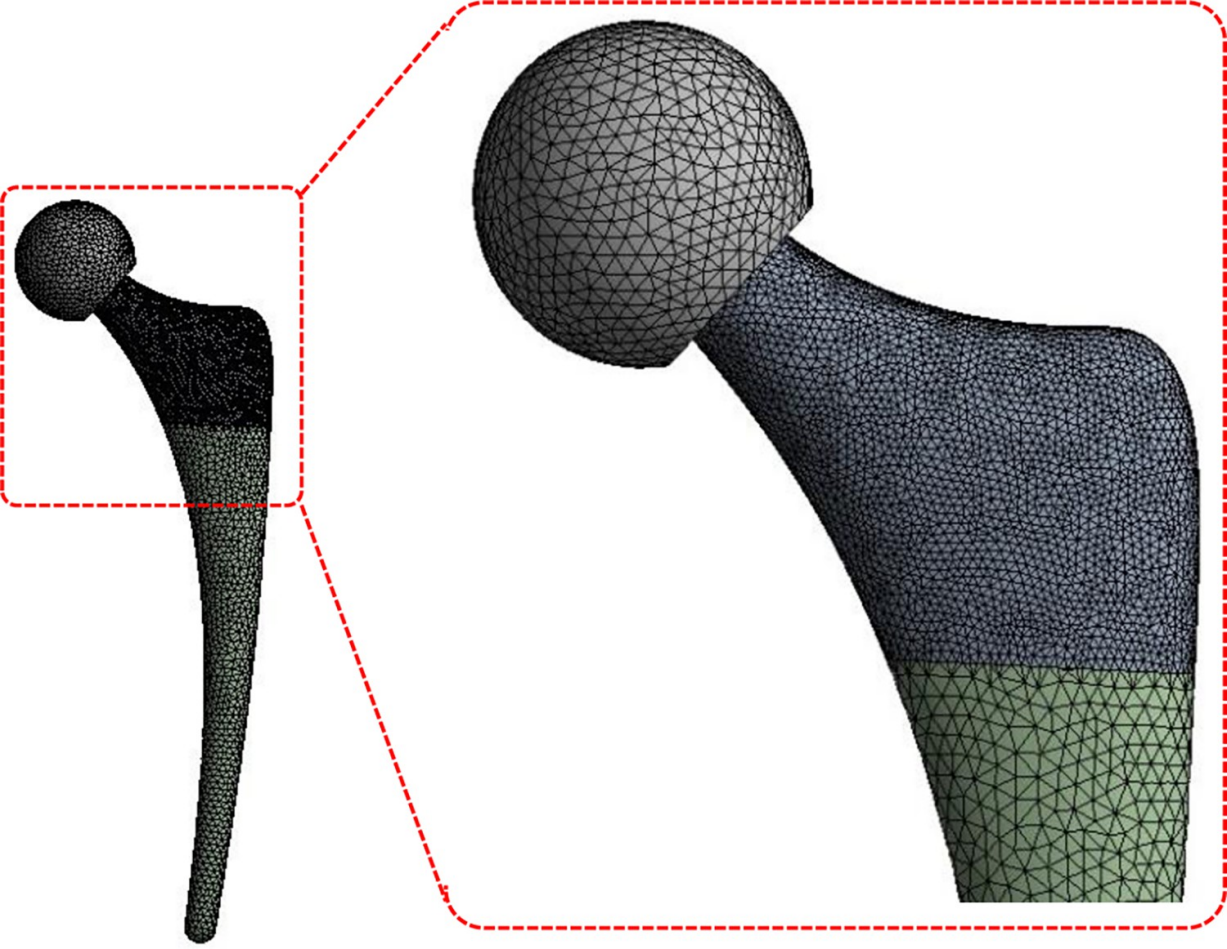
Pictorial representation of mesh 3 utilized for FE analysis.

Fig 3 provides a pictorial representation of the relative grid density in different portion of the hip implant for Mesh 3. This mesh is utilized for all simulations reported in this paper.

For the selected mesh 3, the neck regions is meshed with a body sizing of 1.33 mm whereas rest of the stem and acetabular cup is meshed with a body sizing of 2 mm. Transient structural analysis is conducted for the mesh sensitivity study with load case representing the walking activity and problem constraints explained in the proceeding section. The equivalent Von Mises stress mathematically represented as:

$$\sigma_{e}={\left[\frac{{\left(\sigma_{1}-\sigma_{2}\right)}^{2}+{\left(\sigma_{2}-\sigma_{3}\right)}^{2}+{\left(\sigma_{3}-\sigma_{1}\right)}^{2}}{2}\right]}^{1/2}$$ (2)

Where σ_1_, σ_2_ and σ_3_ are the maximum, middle and minimum principal stresses respectively. The maximum Von Mises stress is selected as the solution variable for the grid independence check.

#### 2.2.2 Simulation setup

A transient structural analysis was conducted in ANSYS Workbench to accurately model the dynamic loads applied to the implant. The total simulation time for each daily activity was determined from experimental force-time data collected by Bergmann et al. [46] using instrumented hip implants. This ensured temporal correspondence between the applied loads and simulated stresses/displacements. The total simulation duration for each activity was discretized into 150 uniform time steps to produce a converged solution using an implicit time integration scheme. Automatic time stepping was employed to refine the time step adaptively based on monitored changes in response variables. Details of the total simulation time and time step for each activity is shown in Table 3.

**Table 3:** Total time and time step for simulation of different activities.

S. No.	Activity	Total Simulation Time [sec]	Simulation Time Step [sec]
1.	Walking	1.123	0.007
2.	Standup	3.195	0.021
3.	Sit-down	3.628	0.024
4.	Stair Up	1.505	0.010
5.	Stairs Down	1.489	0.010
6.	Knee Bend	5.439	0.036
7.	Jogging	0.737	0.005
8.	Cycling	1.449	0.010

**Fig 4:**
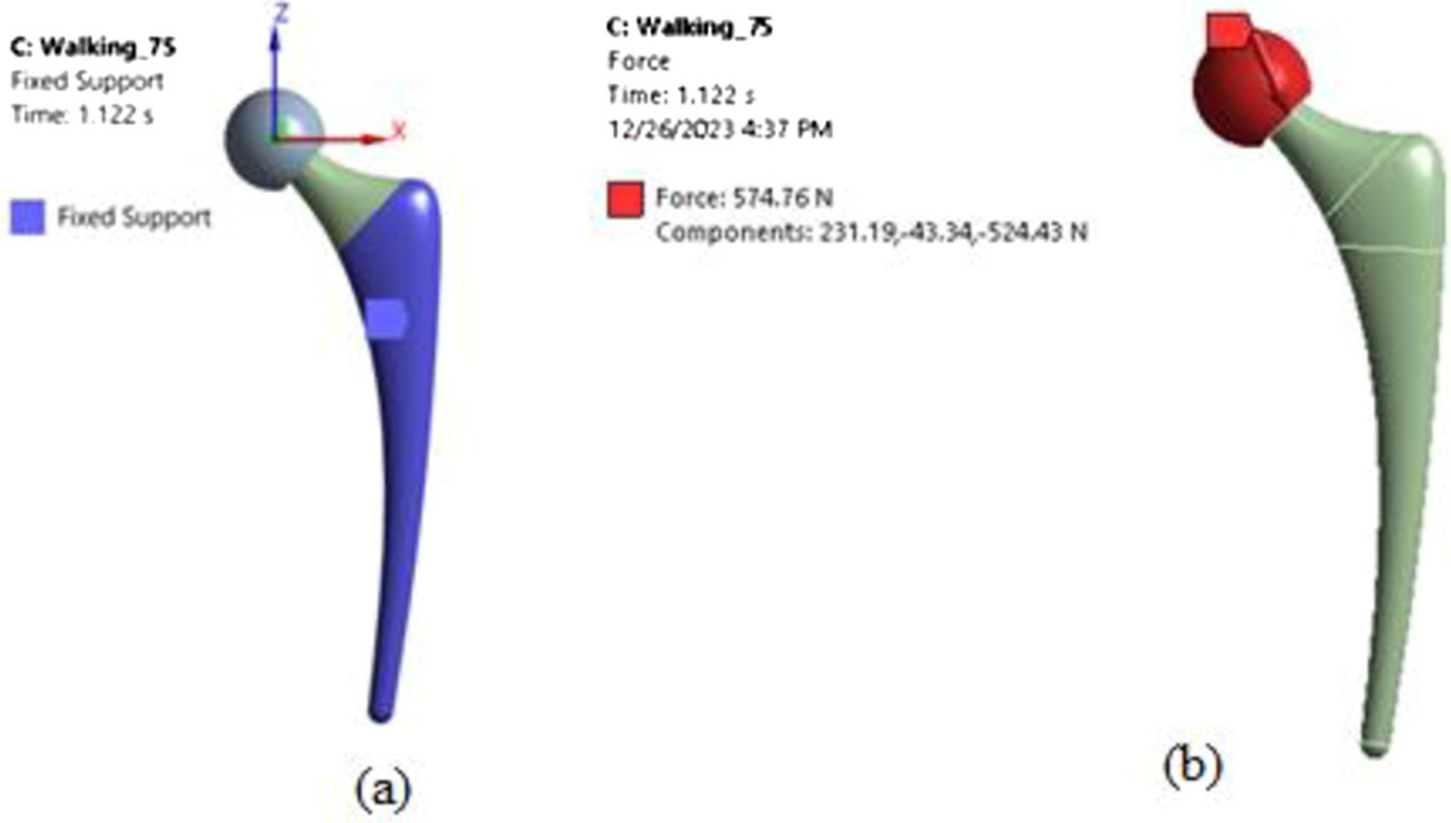
Support and loading conditions for the hip implant model (a) Supports (b) Loading.

Loads applied to the implant were obtained directly from the benchmark experimental dataset for two subject body weights—75 kg and 100 kg. Multiaxial force components resolved in the anatomical coordinate system were imported as discrete load curves. This allowed realistic dynamic loads to be prescribed without approximation or manipulation of the experimental data. A fixed boundary condition (Fig 4) was defined on the implant surfaces embedded in the medullary cavity based on anatomical constraints. All translational and rotational degrees of freedom were constrained to replicate the interfacial fixation between bone and implant achieved in vivo. Rigorous validation was performed to establish mesh and time step independence. Spatial discretization was refined until variations in maximum von Mises stress were less than 5%. Sensitivity analysis established a time step of 0.02 seconds was sufficient for time integration errors to be negligible. Together, this multi-pronged approach ensured simulations accurately captured the stress-time response of the implant under the target applied loads.

**Fig 5:**
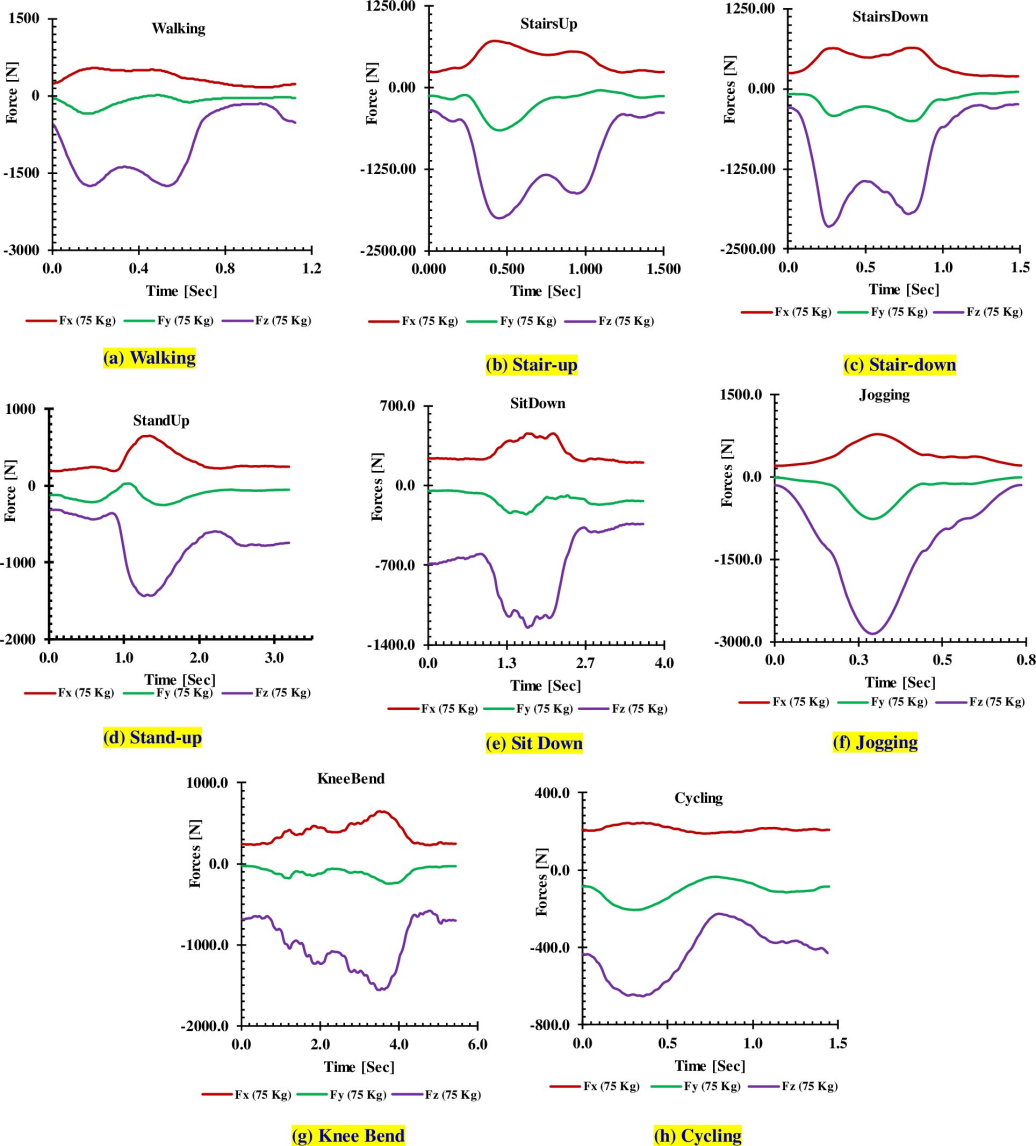
Measured forces for 75 kg.

The applied loads are graphically reproduced in Fig 5 (75 Kg body weight) and from Fig ‎6 (100 Kg body weight) for different activities.

**Fig 6:**
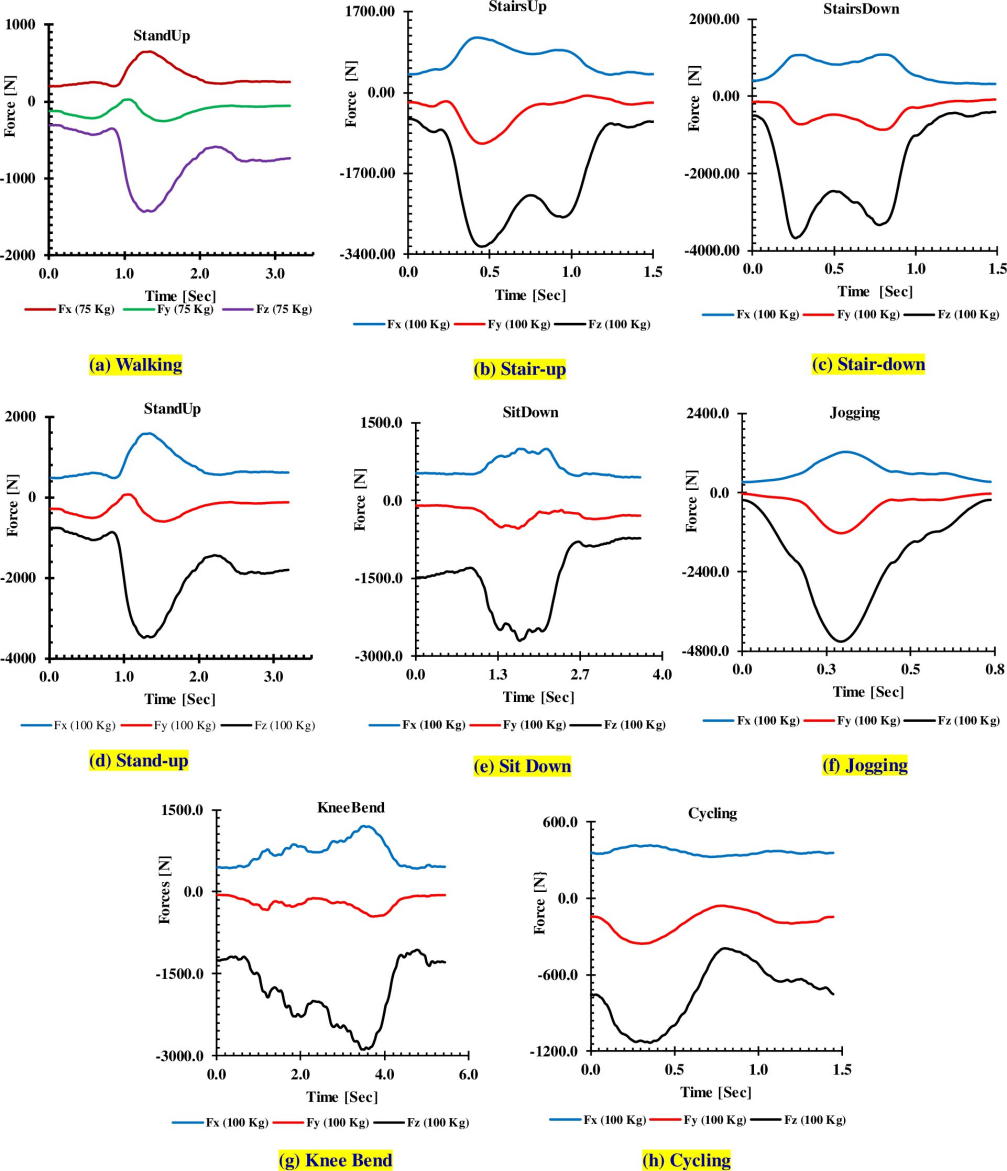
Measured forces for 100 kg.

## 3. Results and discussion

The FE simulation results demonstrated the stress distribution and deformation behavior of the Ti-27Nb hip implant under physiological loading representing different daily activities for 75 kg and 100 kg body weights. The simulated activities included walking, stair ascent/descent, standing up, sitting down, jogging, knee bending and cycling motions, covering a wide range of routine actions with varying load magnitudes and directions. The FE analysis was also conducted for two different conditions of the Ti-27Nb implant–untreated alloy state and after simulated body fluid (SBF) treatment for 816 hours (34 days). This facilitated evaluation of the potential effects of prolonged exposure to physiological fluids on the mechanical integrity and performance of the implant over its service lifetime. The FE results provided detailed insights into the regions of stress concentration within the hip implant components that could potentially be susceptible to damage and failure under repeated cyclic loading. The magnitude of stresses and spatial stress distribution within the implant varied markedly depending on the performed activity and body weight, governed by the resultant joint reaction forces for different load scenarios. Comparison of the peak equivalent stresses against the titanium alloy’s yield strength through generation of safety factor contours enabled assessment of the risk of failure posed by different physiological loading conditions. Lower safety factors would indicate an escalated risk of implant failure. The FE study also assessed the deformation behavior in terms of the total displacements induced within the implant structure under the applied joint loads. Overall, the FE analysis considering a range of simulated physiological loading scenarios provided a more realistic evaluation of the Ti-27Nb hip implant’s biomechanical performance when subjected to the complex loading environment within the human hip joint.

### 3.1 Stress distribution and concentration regions

**Fig 7:**
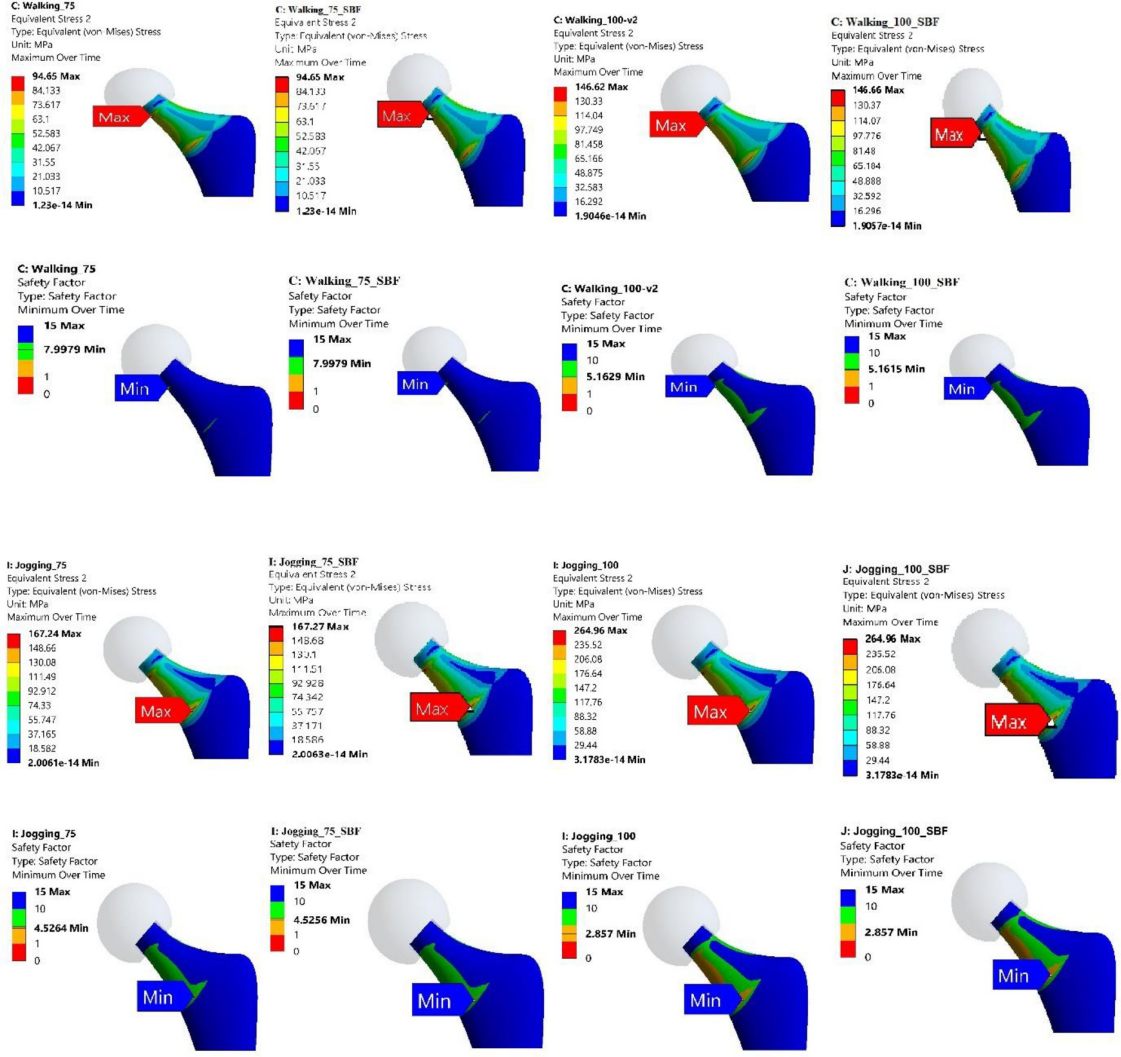
Maximum von Mises Stress and Factor of Safety (FOS) for untreated and simulated body fluid (SBF) treated Ti-27Nb.for walking and jogging activities having body weight of 75 and 100 kg.

**Fig 8:**
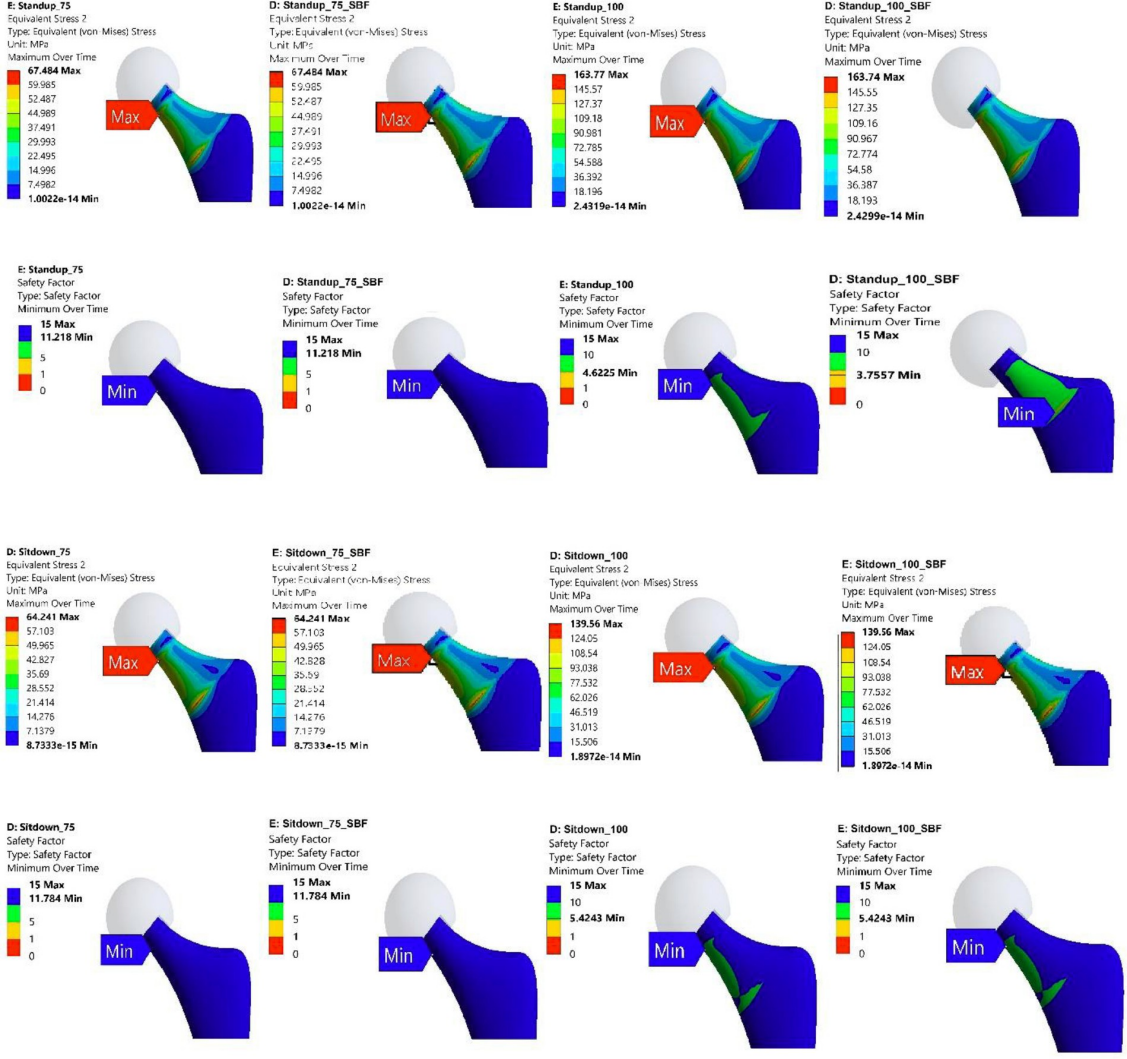
Maximum von Mises Stress and Factor of Safety (FOS) for untreated and simulated body fluid (SBF) treated Ti-27Nb.for standup and sit down activities having body weight of 75 and 100 kg.

**Fig 9:**
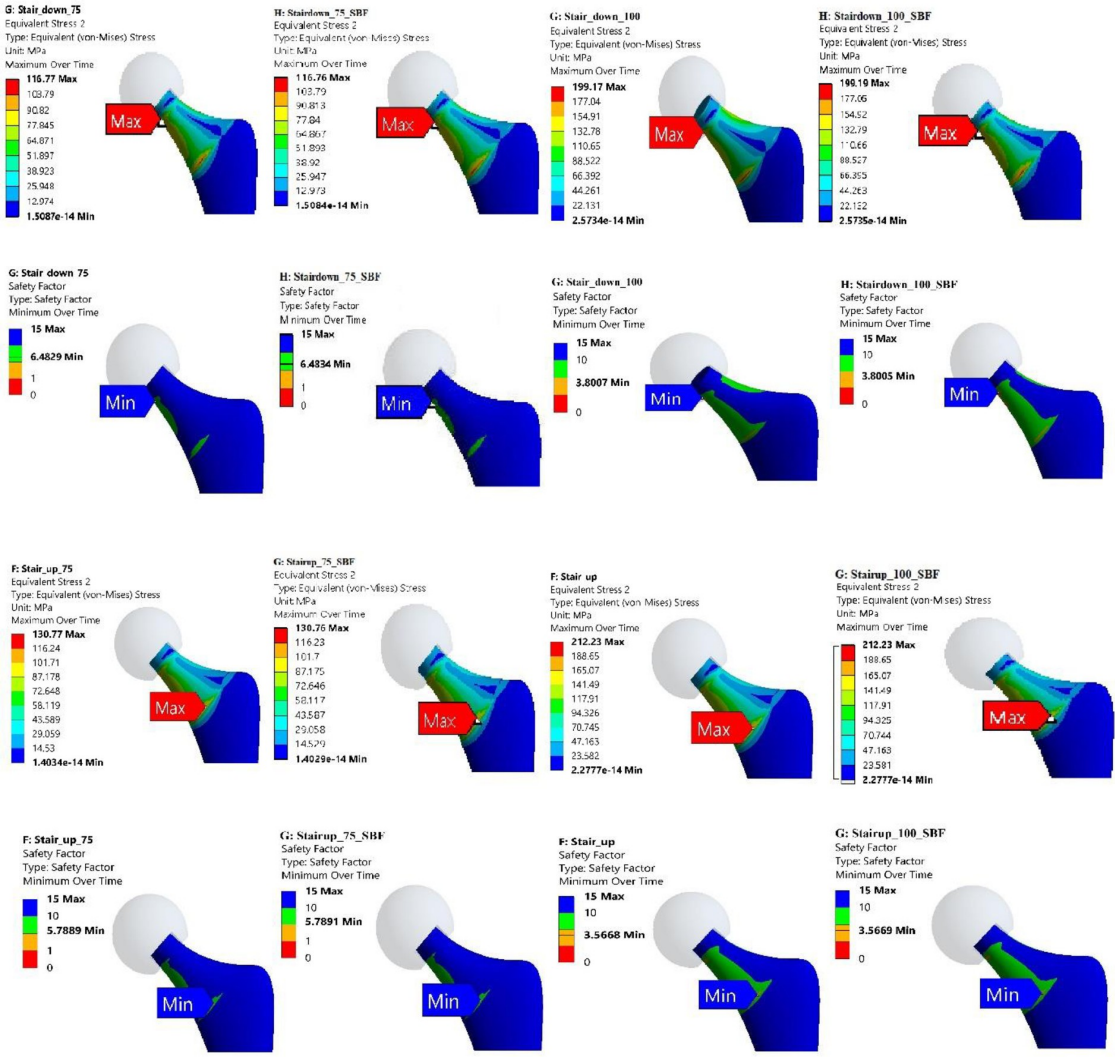
Maximum von Mises Stress and Factor of Safety (FOS) for untreated and simulated body fluid (SBF) treated Ti-27Nb for stair down and stair up activities having body weight of 75 and 100 kg.

**Fig 10:**
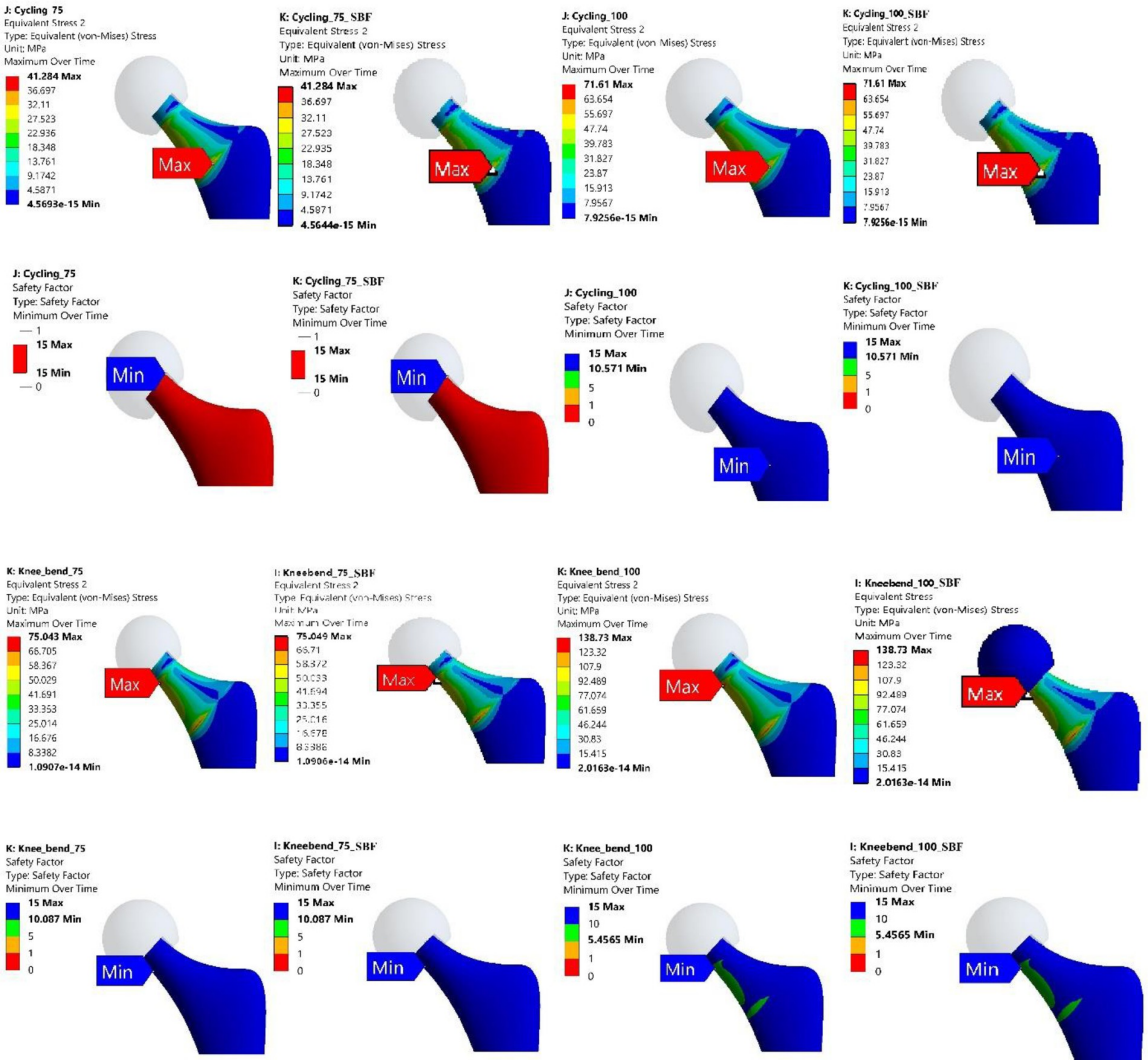
Maximum von Mises Stress and Factor of Safety (FOS) for untreated and simulated body fluid (SBF) treated Ti-27Nb.for cycling and knee bend activities having body weight of 75 and 100 kg.

The FE analysis provided detailed insights into the stress distribution and regions of concentration within the Ti-27Nb alloy hip implant when subjected to the various simulated daily activities. Evaluation of the von Mises stress contour plots revealed that for both 75 kg and 100 kg body mass cases, the localization of peak stresses consistently occurred in the neck region of the hip implant (Figs 7–10). This finding was consistent irrespective of whether the Ti-27Nb implant was in the as-fabricated untreated state or after simulated long-term exposure to body fluid (SBF treated).

The consistent presence of maximum stresses in the implant neck region can be primarily attributed to its relatively smaller cross-sectional area compared to other sections such as the stem and acetabular cup. Based on fundamentals of mechanics of materials, regions with smaller cross-sectional areas along the load path are subjected to higher stresses owing to the reduced area over which to distribute the applied loads. The neck region being narrower than both the stem below it and the femoral head above it, experiences elevated stresses which get further concentrated at certain locations based on the loading directionality.

Although the exact location of the peak von Mises stresses within the neck region shifted between the upper circular section and lower elliptical end adjoining the stem, the maximum stresses remained confined to the neck zone for all simulated daily activities. This clearly highlighted that the neck is the most critical region of the hip implant most susceptible to damage and failure under repeated cyclic loading over long-term implantation.

**Fig 11:**
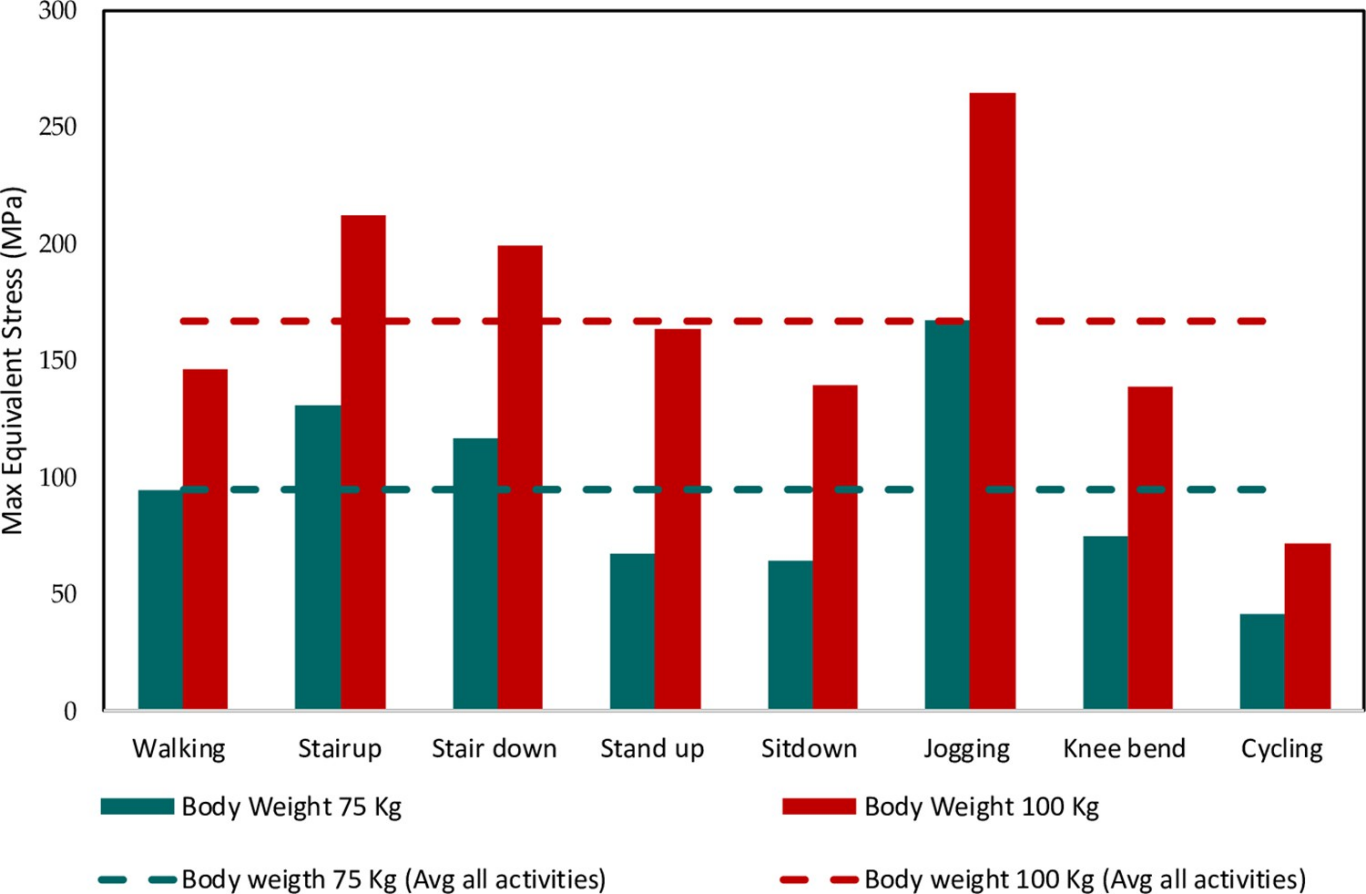
Von Mises stress during different activities of 75 and 100 Kg body weight.

Among the different daily activities simulated through the FE analyses, the stair climbing, jogging and stand-up motions induced the highest von Mises stress magnitudes exceeding the average values obtained across all the activities (Fig 11). For stair ascent and descent motions, the high sideways or medial-lateral forces applied on the hip joint led to elevated localized stresses developing in the implant neck. The neck experiences bending under the medio-lateral load vector, resulting in high stresses on the sides. The stand-up motion also applies larger loads in the medio-lateral direction compared to other motions, generating bending stresses in the implant neck. For jogging, the peaks stresses can be attributed to the dynamic impact loading and larger load magnitudes arising during the high intensity activity, which gets concentrated in the neck’s smaller area.

The FE results showed that the peak von Mises stresses in the implant neck increased significantly for the 100 kg body weight condition compared to the 75 kg case across all simulated daily activities (Fig 11). The maximum von Mises stress values were found to be between 1.2 to 1.8 times higher on average for the heavier 100 kg person than the 75 kg subject across different motions. This trend clearly highlighted the strong influence of higher joint reaction forces occurring due to greater body mass on the resultant stresses induced in the hip implant. Individuals with higher body weight would apply larger forces on the hip joint during activities due to greater inertial effects, gravitational forces and moments, which directly elevate the stresses experienced by the implant. This finding agrees with previous gait analysis studies which have reported markedly increased hip contact forces for obese individuals compared to normal weight subjects during walking and stair climbing activities [75–79].

The FE prediction of elevated stresses and lower safety factors with higher 100 kg body weight corroborates past clinical findings which have associated obese patient populations with increased incidence of implant failure following total hip arthroplasty procedures [79–81]. The excessive stresses beyond optimal levels can accelerate material damage and fracture mechanisms over the long term, compromising implant integrity. This is reinforced by the lower safety factors computed for the 100 kg case, implying greater risk of implant failure when loaded by heavier individuals. Therefore, restricting or limiting certain high intensity activities may be necessary for obese patients with hip replacements based on the FE results. Activity recommendations could help prevent overloading and premature implant failure.

In summary, the FE simulation results clearly identified the neck as the critical region most susceptible to experience damage based on it being the consistent site of peak stresses during all daily activities for both body weights. The neck’s smaller area compared to adjoining sections makes it vulnerable to stress concentrations from multiple load directionalities. Among different motions, the stair ascent/descent, jogging and stand-up activities were found to induce the highest stresses in the neck region which exceeded average values. The neck experienced bending under the dominant medio-lateral loads during these actions. Substantially higher von Mises stresses were predicted with greater 100 kg body weight compared to 75 kg owing to larger joint reaction forces, which agrees with gait studies on obese subjects. The elevated stresses can accelerate material damage and lead to earlier implant failure. The FE results can guide shape and material optimization of the neck region to improve robustness and integrity over long-term use in patients.

### 3.2 Safety factor analysis

The FE study calculated safety factors (FOS) for the Ti-27Nb hip implant under the simulated physiological loading conditions based on the von Mises stress predictions from the FE analyses and the experimentally measured yield strength of the titanium alloy. Evaluation of the safety factors provided crucial insights into the risk of failure posed to the implant by different daily living activities for 75 kg and 100 kg body weights. The safety factor quantitatively indicates the level of structural integrity of a component relative to the applied loads. It is calculated as the ratio of the maximum load or stress that can be sustained before failure to the actual applied load or stress. Typically, a safety factor greater than 1 implies adequate integrity against the applied loads without fracture or excessive damage. The higher the safety factor, the greater is the level of structural reliability.

In this study, the safety factors were computed by taking the ratio of the Ti-27Nb alloy’s yield strength to the maximum von Mises stresses predicted through the FE simulations for different implanted conditions under various activities. The Ti-27Nb alloy’s yield strength value of 733 MPa in the simulated body fluid (SBF) treated condition was used for the most conservative estimates. A quantitative summary of FOS for all activities is provided in Table 4. It is revealed that for both 75 kg and 100 kg body weights, the computed safety factors remained above the minimum desired value of 1 for all simulated daily activities (Table 4). The minimum safety factors were greater than 2.8 across all loading scenarios, despite the conservative yield strength assumption. This indicates that the Ti-27Nb hip implant design evaluated is likely to be structurally safe without risk of fracture when subjected to the range of simulated physiological loads reflecting routine activities. However, the analysis also highlighted considerable reductions in the implant’s safety factors when the body weight of the subject increased from 75 kg to 100 kg. The excessive joint loads associated with higher body mass resulted in increased von Mises stresses under identical activities, thereby lowering the computed safety factors.

**Table 4:** FOS for all activities of 75 and 100 Kg body subject weights (untreated sample).

Activity	Factor of Safety		% Reduction in FOS due to weight increase
	75 Kg. Subject	100 Kg. Subject	
Walking	8	5.1	36.3%
Stair up	5.8	3.5	39.7%
Stair down	6.5	3.8	41.5%
Stand up	11.2	4.6	58.9%
Sit down	11.8	5.4	54.2%
Jogging	4.5	2.8	37.7%
Knee bend	10	5.4	46.0%
Cycling	15	10.5	30.0%

The reduction in safety factors was most severe for jogging, which represented the highest intensity activity with substantial impact loading. The safety factor dropped from 4.5 for 75 kg body weight to 2.8 for 100 kg during jogging. Though not critical, the reduced factor of safety implies potential escalation in failure risk at even higher body weights beyond 100 kg, since the stresses generated would further rise relative to the yield strength. Similarly, low safety factors of 4.6 and 5.4 were predicted for the stand-up and sit-down activities respectively for the 100 kg case in contrast to over 11 for both motions at 75 kg. The substantial reductions of 58.9% and 54.2% in the safety factors for standing up and sitting down loads with greater weight highlights the risks and limitations associated with obesity. This agrees with past clinical findings of markedly higher hip implant failure rates among obese patients [82, 83]. The average safety factor across all activities considered decreased by a considerable 42.9% from 75 kg to 100 kg body weight, indicating significant risks from elevated joint reaction forces resulting from greater body mass loading the hip joint. The results suggest weight-bearing restrictions or limitations in certain intense activities like jogging may be necessary for obese patients with hip replacements to mitigate failure risks arising from excessive stresses. Prescription of optimal activity recommendations and exercise regimens can help prevent overloading.

The stochastic nature of the complex dynamic loads experienced by implants over long durations can lead to material fatigue and accumulated damage even if the maximal stresses themselves are not excessively high [46, 84, 85]. Therefore, while the Ti-27Nb hip implant demonstrated adequate safety factors greater than 1, the reductions with higher weight implies lower endurance limits over the device lifetime. This could potentially translate to earlier onset of fatigue failure. The FE results can guide suitable modifications to the implant shape and geometry design, such as increasing neck diameter and thickness, to improve the structural robustness and integrity thereby providing higher factors of safety under heavier physiological loads. Changes in geometry to minimize stress concentrations may also be warranted based on the FE findings. Additionally, surface treatments to facilitate osseointegration and bone ingrowth could offer better load transfer to the periprosthetic bone, lowering stresses experienced by the implant itself. Enhanced fixation at the bone-implant interface would be highly desirable to mitigate risks of loosening and fatigue failure resulting from excessive micromotion [86–89]. Alternative alloy compositions and material microstructural modifications to increase strength and fatigue resistance should also be explored to bolster implant reliability for heavier patients.

In summary, the comprehensive FE-based safety factor analysis provided vital insights into the reductions in structural integrity margins and escalated failure risks posed by higher body weight loading scenarios. The findings highlight the need for suitable activity recommendations and interventions for obese patients to prevent premature implant failure. The results can guide geometrical and material enhancements of hip implant designs to sufficiently withstand elevated physiological stresses induced by greater body habitus over long-term use.

### 3.3 Treatment effects

The finite element study also evaluated the effects of prolonged simulated physiological fluid exposure on the mechanical integrity of the Ti-27Nb hip implant through comparative analysis between the as-fabricated untreated alloy condition versus treated state after immersion in simulated body fluid (SBF) for 816 hours. As the human body essentially represents a corrosive aqueous medium containing ions, proteins and other constituents, investigating the effects of such environments on the implanted biomaterial’s stability is crucial to determining its long-term performance in vivo. Material degradation over the implant’s lifespan could potentially compromise the implant’s structural integrity and cause premature failures. The FE simulations of the Ti-27Nb hip implant under daily activity loading were therefore conducted for both untreated and SBF treated conditions to understand variations in mechanical response due to physiologically relevant fluid interactions. The results showed no appreciable differences in the maximum von Mises stress values and distribution between untreated and SBF treated states for either 75 kg or 100 kg body mass simulations across all the daily living activities modeled.

The nearly identical von Mises stress profiles imply that prolonged emersion in the simulated biological fluid had negligible effects on the biomechanical behavior and integrity of the Ti-27Nb implant when subjected to physiological loading. No discernible degradation in load-bearing capacity was evident after SBF treatment. Moreover, the computed safety factors which provide insights into the implant’s structural integrity against failure remained unchanged between untreated and SBF exposed conditions. The Ti-27Nb hip implant maintained similar safety factors above the desired minimum value of 1 subsequent to SBF treatment for all activities, further confirming preserved robustness. The minimal effects of SBF exposure corroborate with prior experimental characterization performed by the authors where negligible differences were found in the Ti-27Nb alloy’s mechanical properties of yield strength, ultimate tensile strength and elastic modulus after SBF immersion compared to the untreated state [30, 67, 90]. The mechanical testing also revealed unchanged fatigue crack growth resistance and fracture toughness behavior for Ti-27Nb following prolonged SBF treatment. The high corrosion resistance of titanium alloys in biological environments owing to the stable protective oxide layer renders the Ti-27Nb resistant to degradation via electrochemical or chemical reactions. The lack of harsh free-radicals helps prevent extensive damage over implantation timescales [91, 92]. The FE results affirm retention of strength and resistance to failure which agree with the measured properties.

The finding that simulated physiological conditions for time durations beyond the implant’s expected service lifetime do not adversely impact the Ti-27Nb’s mechanical performance provides strong support for its long-term stability in vivo. This further validates the alloy’s suitability for use in load-bearing total hip arthroplasty applications which demand high endurance over decades without compromised integrity leading to catastrophic failures. The minimal variations in biomechanical response also imply that post-implantation changes in the periprosthetic bone morphology as it remodels and interfaces with the implant are unlikely to drastically influence the implant stresses for this particular alloy system. Gradual adaptive bone changes would produce equivalent effects on untreated and treated implant configurations. The safety factors against yield which predominantly depend on the alloy’s mechanical properties are also expected to remain unaltered over extended implantation, unless other physical damage like cracks or fretting occurs. However, follow-up studies tracking the progressive bone adaptation effects would provide further validation. The superior corrosion resistance of Ti-27Nb compared to conventional cobalt-chromium-molybdenum implants also makes it less susceptible to metal ion release and particle generation which cause adverse tissue reactions and osteolysis. Reduced corrosion and wear implications coupled with stable biomechanical integrity is highly advantageous for hip replacement longevity.

However, the effects of dynamic fatigue damage processes need investigation since the current study was limited to static loading conditions. Physiological cyclic stresses can potentially interact with the in vivo environment over long durations to activate subtle mechano-chemical degradation mechanisms [93, 94]. The initial material microstructure and surface characteristics may also evolve over years of activity. Therefore, the next phase of analysis should incorporate realistic dynamic loading profiles from gait measurements to simulate walking cycles over multiple years along with solution effects to conclusively ascertain the alloy’s fatigue resistance and perennial integrity post-implantation. Nevertheless, the present study’s finding that simulated physiological fluid interactions do not adversely impact the Ti-27Nb hip implant’s mechanical response and safety factors under static loading provides useful insights into its long-term stability as a prosthesis material. The results support the alloy’s implementation to mitigate risks of premature failures resulting from material degradation.

In summary, comparative analysis between untreated and physiological fluid treated states via FE simulation demonstrated negligible effects on Ti-27Nb’s biomechanical performance, which agrees with the alloy’s high corrosion resistance properties. This further validates its long-term integrity and functionality as a reliable bearing surface material for total hip arthroplasty applications. However, follow-up dynamic fatigue studies would provide more conclusive validation.

## 4. Conclusions

In this study, finite element analysis was performed to investigate the stress responses and safety factors of a hip implant made of Ti-27Nb alloy under various routine daily activities and different body weights. Contour plots of maximum von Mises stress and factor of safety were presented. The following key conclusions were drawn:

The location of maximum von Mises stress was consistently in the neck region for all activities and remained unaffected by SBF treatment. However, its precise location varied depending on the relative contribution of load components. Activities with higher x- and/or y-load components shifted the peak stress location to the bottom elliptical neck section.

Jogging produced the highest von Mises stress of 265 MPa whereas cycling yielded the lowest of 72 MPa. Stair climbing/descending and jogging also exceeded the average stress level across activities. Maximum stresses substantially increased with body weight, confirming its influence on implant damage accumulation.

Despite values lying in the safe zone, safety factors reduced significantly with rising body weight, implying increased failure risks for obese patients. Stand-up and sit-down experienced the maximum 58.9% and 54.2% decrements from 75 to 100 kg, respectively. On average, weight gain decreased safety factors by 42.9%.

Comparative analyses between untreated and long-term SBF treated implant states revealed negligible biomechanical variations altering safety factors by only 2% maximally for SBF treatment. This validates Ti-27Nb alloy’s inherent resistance to in vivo degradation under functional loadings.

### 4.1 Future recommendations

- While this study provided valuable insights, further research is warranted to more comprehensively evaluate Ti-27Nb hip implants. Potential areas that merit investigation include:
- Repeated/fatigue loading analyses accounting for varying activities and body weights to assess long-term durability under realistic service conditions.
- Experimental validation of finite element predictions through strain gauge/pressure sensor instrumented implant prototypes and cadaveric/animal trials.
- Parametric studies exploring the effects of implant design modifications like cellular architectures, surface coatings on stress distributions and implant fixity.
- Multiscale analyses incorporating intracortical bone remodeling responses to modeling results for a more consolidated implant-bone system level assessment.
- Probabilistic approaches involving statistical distributions of patient/surgical variables to determine likely failure ranges in clinical populations.
- In vitro/in vivo biological assessments to confirm long-term osseointegration and healing capability of SBF treated Ti-27Nb.

Rigorous validation and addressing the above aspects would translate the current advances into tangible clinical practices, taking full advantage of Ti-27Nb alloy’s potential as a next-generation hip implant material.
